# Supplementary material for: PERK activation mitigates tau pathology in vitro and in vivo
Source: EMBO Mol Med. 2017 Feb 1;9(3):371–84. doi: 10.15252/emmm.201606664 (PMC5331260; doi:10.15252/emmm.201606664)
Supplement: Supplementary file 1 — Appendix [file EMMM-9-371-s001.pdf]

PERK activation mitigates tau pathology *in vitro* and *in vivo*

Julius Bruch[1,2,3,#], Hong Xu[1,2,3,#], Thomas W. Rösler[1,2,#], Anderson De Andrade[1,3], Peer-Hendrik Kuhn[4,5], Stefan F. Lichtenthaler[3,4,6], Thomas Arzberger[7], Konstanze F. Winklhofer[3,8], Ulrich Müller[9], Günter U. Höglinger[1,2,3,*]

#These authors contributed equally to the work.

*Corresponding author

Appendix Table of Contents:

Appendix Figures

Appendix Figure S1. Lentiviral overexpression of tau isoforms *in vitro*.

Appendix Figure S2. PERK activation prevents annonacin-induced neurofilament dephosphorylation.

Appendix Figure S3. PERK activation *in vivo*.

Appendix Figure S4. Silencing efficacy of *NFE2L2* siRNA.

Appendix Table S1. Details of substances used for treatment

Appendix Table S2. Overview of plasmids used for cloning

Appendix Table S3. Primers and restriction enzymes used for cloning of vector plasmids

Appendix Table S4. Overview of human tissue samples used

Appendix Table S5. Overview of antibodies used

Appendix Table S6. *P*-values of figures

References

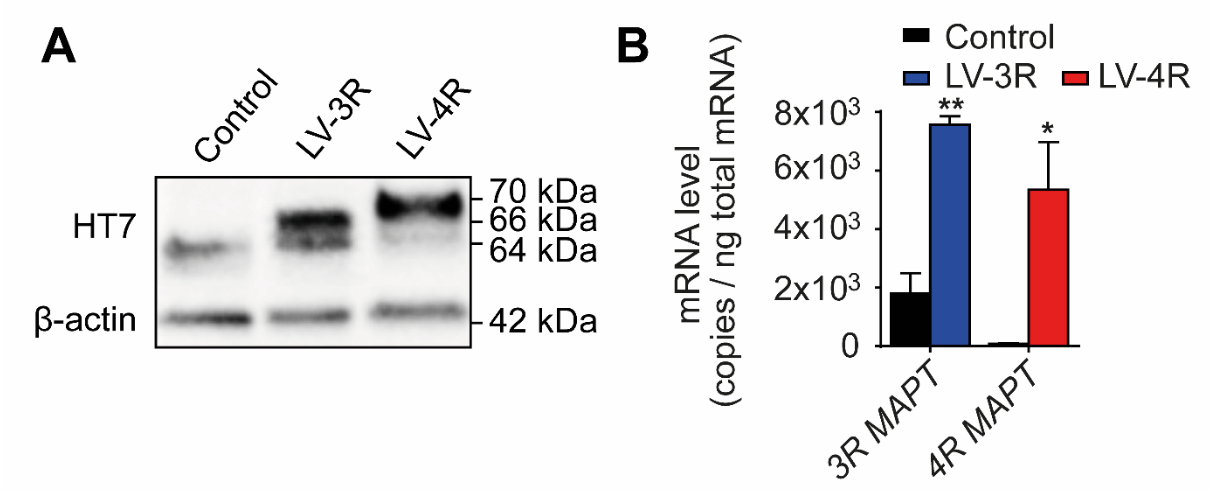

Appendix Figure S1. Lentiviral overexpression of tau isoforms *in vitro*.

A Western blot of total tau protein (HT7 antibody) showing the overexpression of different isoforms in LUHMES neurons after 8 days of differentiation compared to untreated (control) neurons or neurons incubated with lentiviruses encoding 3R (LV-3R) or 4R (LV-4R) tau. The predominant endogenous isoform at this stage of differentiation is the 3R subisoform of approximately 64 kDa. The 3R2N isoform overexpressed by LV-3R runs at 66 kDa, and the molecular weight of the 4R2N isoform overexpressed by LV4R is about 70 kDa. Actin was used as loading control.

B RT-qPCR of 4R and 3R tau mRNAs in 8 days old LUHMES neurons, treated as in (A) (n = 3 per condition). Data are mean + SEM. Statistical analysis was Student's t-test; $^{*}P < 0.05$, $^{**}P < 0.01$ vs. control.

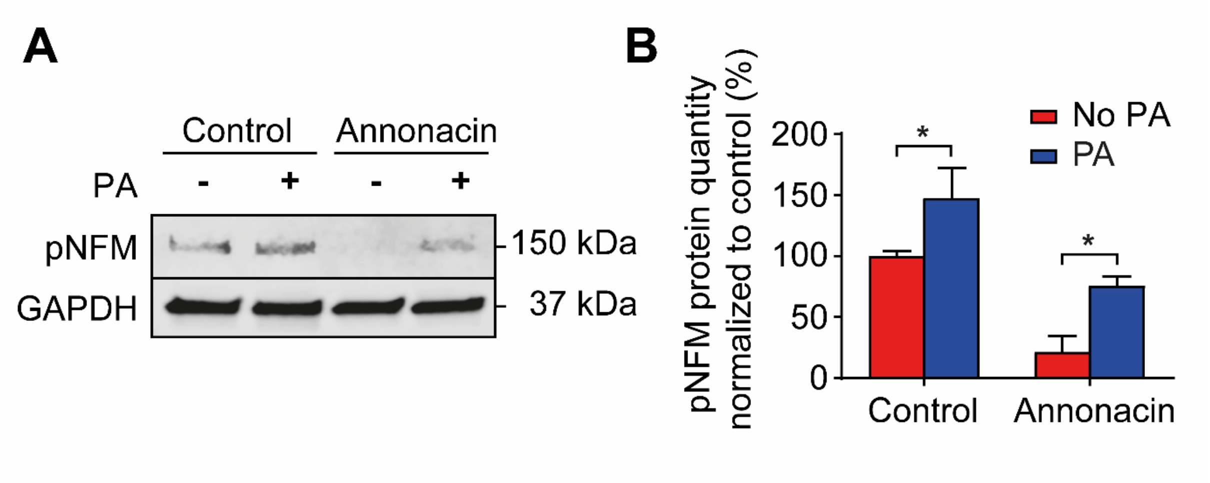

Appendix Figure S2. PERK activation prevents annonacin-induced neurofilament dephosphorylation.

A Representative Western blot of LUHMES neurons treated with or without annonacin (25 nM) and with or without the PERK activator (PA, 200 nM). The antibodies depicted phosphorylated neurofilament-medium polypeptide (pNFM) and GAPDH as loading control.

B Densitometric analysis of Western blots described in (A) ($n = 3$), normalized to the loading control and to untreated control cells. Data are mean + SEM. Statistical analysis was ANOVA followed by Newman-Keuls multiple comparison test. $*P < 0.05$.

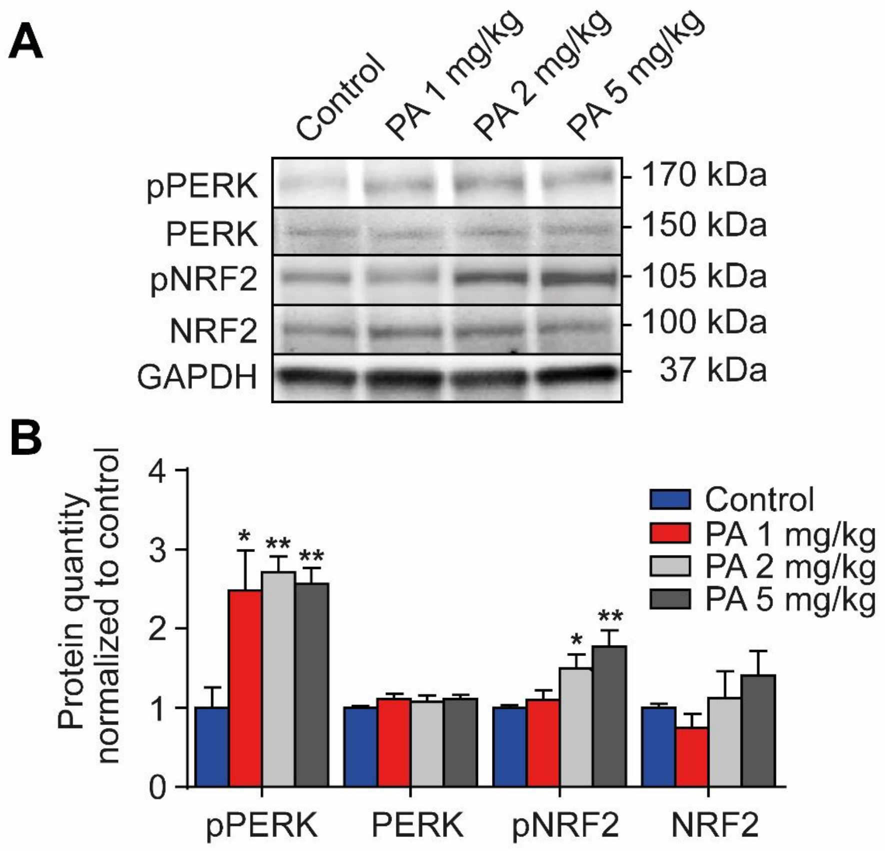

Appendix Figure S3. PERK activation *in vivo*.

A 15-week-old wildtype mice were injected i.p. with different doses of PERK activator (PA; 1, 2 or 5 mg/kg) once daily for 3 days. Untreated mice were used as controls. Representative Western blots of whole brain homogenates immunostained with antibodies against phosphorylated PERK (pPERK), total PERK, phosphorylated NRF2 (pNRF2) and total NRF2. GAPDH was used as loading control.

B Densitometric analysis of Western blot (A), normalized to untreated controls (n = 3). Data are mean + SEM. Statistical analysis was one-way ANOVA with post-hoc Fisher's LSD test. *P < 0.05, **P < 0.01 vs. control.

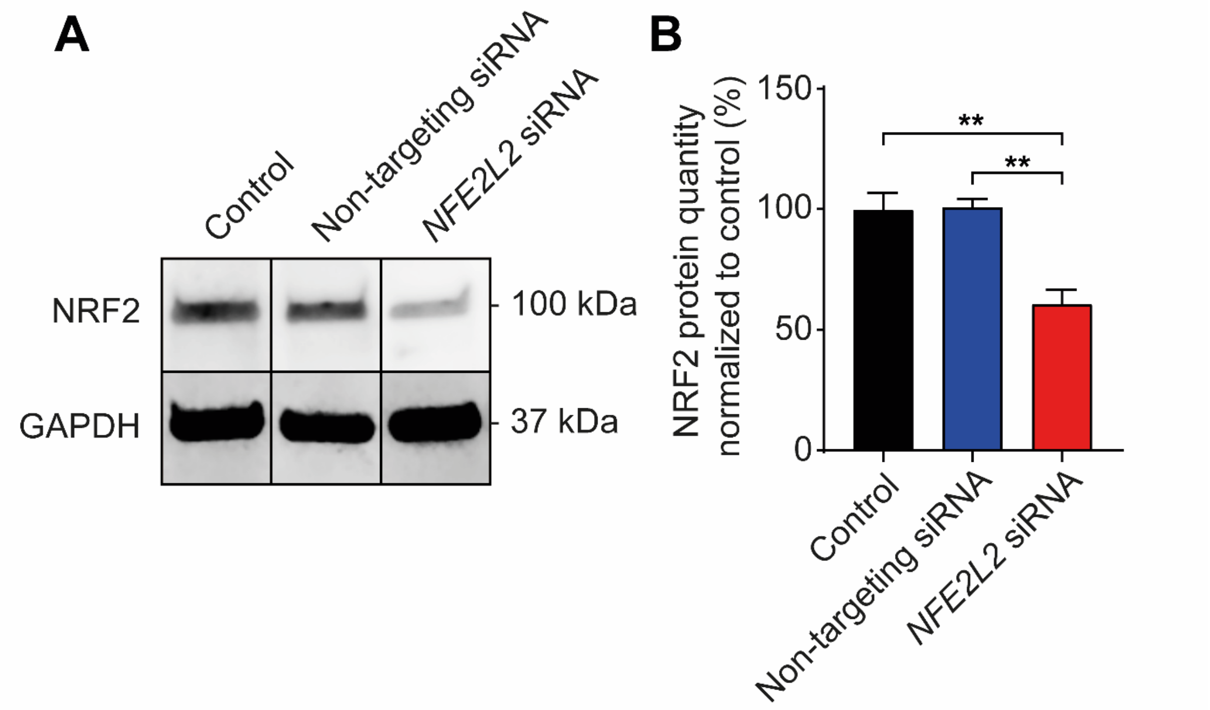

Appendix Figure S4. Silencing efficacy of *NFE2L2* siRNA.

A Representative Western blots of LUHMES neurons untreated or treated with 50 nM non-targeting siRNA or *NFE2L2* siRNA. The antibodies depicted nuclear factor (erythroid-derived 2)-like 2 (NRF2) and GAPDH as loading control.

B Densitometric analysis of Western blots described in (A) ($n = 3$), normalized to the loading control and to untreated control cells. Data are mean + SEM. Statistical analysis was one-way ANOVA followed by Tukey’s post-hoc test. **$P < 0.01$.

Appendix Table S1

Details of substances used for treatment

Substance name	Source	Dissolving and dilution	Treatment concentration*	Treatment duration*
Annonacin	Extracted from Annona muricata L. fruits by Pierre Champy, Univ. Paris–Sud, France	Dissolved in DMSO (AppliChem) to 1 mM, diluted further with medium	25 nM	48 h (days 8 – 10 post differentiation)
PERK activator (CCT020312) (Stockwell et al, 2012)	Merck Millipore, Billerica, MA, USA	Dissolved in DMSO to 10 mM, diluted further with medium	200 nM	48 h (days 8 – 10 post differentiation)
PERK inhibitor (GSK2606414)	Toronto Research Chemicals, North York, ON, Canada	Dissolved in DMSO to 10 mM, diluted further with medium	300 nM	48 h (days 8 – 10 post differentiation)
Thapsigargin	AppliChem, Darmstadt, Germany	Dissolved in DMSO to 10 mM, diluted further with medium	30 nM	48 h (days 8 – 10 post differentiation)
DL-sulforaphane-N-acetyl-L-cysteine	Cayman Chemical (16098)	Dissolved in DMSO to 100 mM, diluted further with medium	100 nM	48 h (days 8 – 10 post differentiation)
Stealth NFE2L2 siRNA	5843822, Thermo Fisher Scientific	RNAse free water (Thermo Fisher Scientific)	50 nM	post differentiation day 4
Stealth non-targeting siRNA (medium GC duplex #2)	465372, Thermo Fisher Scientific	RNAse free water (Thermo Fisher Scientific)	50 nM	post differentiation day 4

*unless indicated otherwise

Appendix Table S2

Overview of plasmids used for cloning

Gene Insert	Species	Full plasmid name	Source	Reference
MAPT 2N3R	Human	pRK172/htau39	Eva-Maria Mandelkow, DZNE Bonn, Germany	
MAPT 2N4R	Human	pNG2 htau40	Eva-Maria Mandelkow, DZNE Bonn, Germany	
Eif2ak3	Mouse	PERK.WT.9E10.pCDNA.amp	David Ron, University of Cambridge, UK (via Konstanze Winklhofer, Ruhr Universität Bochum Bochum, Germany)	(Bouman et al, 2011)
n.a.	n.a.	FU-ΔZeo	Stefan Lichtenthaler, DZNE Munich, Germany	(Kuhn et al, 2010)
mCherry	Aequorea victoria	mCherry/FU- ΔZeo	Stefan Lichtenthaler, DZNE Munich, Germany	

Appendix Table S3

Primers and restriction enzymes used for cloning of vector plasmids

Original plasmid	Forward primer	Reverse primer	Restriction enzymes
pRK172/htau39	GATCTCTAGAATCACAAA CCCTGCTTGGCCAG	GATCGGATCCGATATACATA TGGCTGAGCC	BamH1-HF, Xba1
pNG2 htau40	GATCTCTAGAATCACAAA CCCTGCTTGGCCAG	GATCGGATCCGGAGATATAC ATATGGCTGAGCC	BamH1-HF, Xba1
PERK.WT.9E10. pCDNA.amp	caggtcgactctagagGCGATGTC TGCACAAGGC	cgataagcttgatatcgGCCAGGCAG TGGCGTGTA	Gibson Assembly Mastermix

Appendix Table S4

Overview of human tissue samples used

Case number	Diagnosis	Age at death	Sex	Postmortem time (h)
C1	Control	78	Female	8
C2	Control	77	Female	10
C3	Control	77	Female	20
C4	Control	73	Female	17
C5	Control	66	Female	27
C6	Control	74	Male	33
P1	PSP	62	Female	9
P2	PSP	76	Male	12
P3	PSP	76	Female	10
P4	PSP	75	Male	12
P5	PSP	67	Male	40
P6	PSP	69	Male	12
P7	PSP	78	Female	30

Appendix Table S5

Overview of antibodies used

Antigen	Clone	Species	Concentration	Source
PERK	D11A8	Rabbit	1:1000 (WB)<br>1:100 (IP)	Cell Signaling Technology
pT980-PERK	16F8	Rabbit	1:1000	Cell Signaling Technology
p-Ser-396 PHF Tau	AD2	Mouse	1:2000	BioRad
4 repeat Tau	1E1/A6	Mouse	1:333	Merck Millipore
3 repeat Tau	8E6/C11	Mouse	1:1000	Merck Millipore
p-Ser-202 Tau	CP13	Mouse	1:500	Peter Davies, Albert Einstein College, NY, USA
Conformationally changed Tau	MC1	Mouse	1:333	Peter Davies, Albert Einstein College, NY, USA
Total human tau	HT7	Mouse	1:1000	Thermo Fisher Scientific
EIF2A	polyclonal	Rabbit	1:1000	Cell Signaling Technology
pS51-EIF2A	D9G8	Rabbit	1:1000	Cell Signaling Technology
NRF2	ab62352	Rabbit	1 : 1 000	Abcam
pS40-NRF2	EP1809Y	Rabbit	1: 1000	GeneTex (Irvine, CA, USA)
pNFM	RMO55	Mouse	1: 1000	Milllipore
HO-1	HO-1-1	Mouse	1: 250	Abcam

Appendix Table S6

P-values of figures

Figure		Comparison	Significance symbol	*P*-Value
**Figure 1B**	pPERK	Control vs. PSP	*	0.0355
	PERK	Control vs. PSP	*	0.0280
	pEIF2A	Control vs. PSP	*	0.0353
	EIF2A	Control vs. PSP	**	0.0018
	pNRF2	Control vs. PSP	**	0.0096
	NRF2	Control vs. PSP	***	0.0001
**Figure 1D**	pPERK	Control vs. THG	***	0.0001
		Control vs. ANN	***	0.0005
	PERK	Control vs. THG	*	0.0100
		Control vs. ANN	**	0.0031
	pEIF2A	Control vs. THG	***	0.0001
		Control vs. LV-4R	**	0.0085
	EIF2A	Control vs. THG	**	0.0099
		Control vs. ANN	**	0.0043
		Control vs. LV-4R	*	0.0318
**Figure 2A**	ANN 12.5 nM	PA vs. Control	*	0.0155
	ANN 25 nM	PA vs. Control	*	0.0262
	ANN 50 nM	PA vs. Control	***	<0.0001
	ANN 100 nM	PA vs. Control	*	0.0200
**Figure 2B**	ANN 12.5 nM	PA vs. Control	**	0.0013
	ANN 12.5 nM	PI vs. Control	++	0.0013
	ANN 25 nM	PA vs. Control	**	0.0062
	ANN 50 nM	PA vs. Control	***	<0.0001
**Figure 2C**	MTT	LV-mCh vs. LV-4R	###	0.0004
		LV-4R vs. LV-4R + PA	###	0.0007
**Figure 2D**	ATP	LV-mCh vs. LV-4R	###	<0.0001
		LV-mCh vs. LV-4R + PA	###	<0.0001
		LV-4R vs. LV-4R + PA	###	<0.0001
		LV-4R vs. LV-4R + PI	###	<0.0001
**Figure 2G**	Cell number	Control vs. LV-4R	##	0.0093
		Control vs. ANN	#	0.0427
		LV-4R vs. LV-4R + PA	#	0.0355
		ANN vs. ANN + PA	##	0.0014
		ANN + PA vs. ANN + PI	##	0.0048
**Figure 3B**	MC1	Control vs. ANN	*	0.0280
		ANN vs. ANN + PA	##	0.0014
	CP13	ANN vs. Control	*	0.0289
		ANN vs. ANN + PA	#	0.0355
	AD2	ANN vs. Control	*	0.0191
		ANN vs. ANN + PA	#	0.0139
**Figure 3D**	4R tau	Control vs. ANN	***	<0.0001
		Control vs. ANN + PA	**	0.0026
		ANN vs. ANN + PA	###	<0.0001
**Figure 3E**	*4R MAPT*	Control vs. ANN	***	<0.0001
		ANN vs. ANN + PA	###	<0.0001
**Figure 3F**	*SRSF2*	Control vs. ANN	***	<0.0001
		ANN vs. ANN + PA	#	0.0131
**Figure 3H**	CP13	Control vs. LV-4R	***	0.0009
		LV-4R vs. LV-4R + PA	##	0.0057
	AD2	Control vs. LV-4R	*	0.0113

Figure		Comparison	Significance symbol	*P*-Value
		Control vs. LV-4R + PA	*	0.0236
	HT7	Control vs. LV-4R	*	0.0136
		Control vs. LV-4R + PA	*	0.0152
**Figure 4B**	pPERK	WT + PA vs. WT + NS	*	0.0143
	pNRF2	WT + PA vs. WT + NS	*	0.0493
**Figure 4D**	MC1	P301S + PA vs. P301S + NS	*	0.0471
	CP13	P301S + PA vs. P301S + NS	*	0.0405
	AT180	P301S + PA vs. P301S + NS	*	0.0404
	HT7	P301S + PA vs. P301S + NS	*	0.0205
**Figure 4F**	HT7	P301S + PA vs. P301S + NS	*	0.0250
	MC1	P301S + PA vs. P301S + NS	*	0.0430
	CP13	P301S + PA vs. P301S + NS	*	0.0265
	AT180	P301S + PA vs. P301S + NS	*	0.0269
**Figure 5A**	Latency to find platform	WT + NS vs. P301S + NS	*	0.0412
		P301S + NS vs. P301S + PA	#	0.0287
**Figure 5C**	Spine density	WT + NS vs. P301S + PA	#	0.0293
		WT + NS vs. P301S + NS	***	0.0002
		P301S + NS vs. P301S + PA	##	0.0073
**Figure 5D**	Latency to fall	WT + NS vs. P301S + PA	#	0.0475
		WT + NS vs. P301S + NS	***	<0.0001
		P301S + NS vs. P301S + PA	###	<0.0001
**Figure 5E**	Motoneuron number	WT + NS vs. P301S + NS	*	0.0282
		P301S + NS vs. P301S + PA	#	0.0319
**Figure EV1B**	pPERK	P301S 2m vs. WT 6m	*	<0.05[a]
		P301S 6m vs. WT 6m	*	<0.05[a]
		P301S 2m vs. P301S 6m	**	<0.01[a]
	PERK	P301S 6m vs. WT 6m	*	<0.05[a]
		P301S 2m vs. P301S 6m	*	<0.05[a]
	pEIF2A	P301S 2m vs. WT 6m	*	<0.05[a]
		P301S 6m vs. WT 6m	*	<0.05[a]
	pNRF2	P301S 6m vs. WT 6m	***	<0.0001[a]
		P301S 2m vs. P301S 6m	***	<0.0001[a]
**Figure EV2A**	MTT	PA 10 µM vs. Control	*	0.0264
**Figure EV2E**	pEIF2A	PA vs. Control	*	0.0486
		PI vs. Control	*	0.0452
	pNRF2	PA vs. Control	*	0.0378
		PI vs. Control	*	0.0387
		ANN vs. Control	*	0.0297
		ANN + PA vs. Control	*	0.0135
		ANN + PI vs. Control	*	0.0375
		ANN vs. ANN + PA	##	0.0025
**Figure EV2G**	pEIF2A	LV-4R vs. Control	*	0.0212
		LV4R + PA vs. Control	*	0.0421
		LV-4R + PI vs. Control	**	0.0099
		LV-3R vs. Control	*	0.0164
		LV-3R + PA vs. Control	*	0.0200
		LV-3R + PI vs. Control	*	0.0348
		LV-4R vs. LV-4R + PI	##	0.0073
	pNRF2	LV-4R vs. LV-4R + PA	#	0.0493
**Figure EV3**	Neuritic network density	LV-4R vs. Control	***	<0.0001
		LV-4R vs. LV-4R + PA	***	<0.0001
		LV-4R vs. LV-4R + PI	***	0.0002
		ANN vs. Control	***	<0.0001
		ANN vs. ANN + PA	**	0.0015
		ANN vs. ANN + PI	***	<0.0001
		ANN + PA vs. ANN + PI	***	<0.0001

Figure		Comparison	Significance symbol	*P*-Value
**Figure EV4B**	HO-1	PA vs. Control	*	0.0237
**Figure EV4C**	LV-4R	No SFN-NAC vs. SFN-NAC	*	0.0458
**Figure EV4D**	LV-4R	*NFE2L2* siRNA vs. No siRNA	***	<0.0001
		NFE2L2 siRNA vs. Non-targeting siRNA	***	0.0002
**Figure EV5B**	*4R MAPT*	ANN vs. Control	***	0.0004
		ANN vs. ANN + LV-PERK	##	0.0027
**Figure EV5C**	MTT	ANN vs. Control	***	<0.0001
		ANN vs. ANN + LV-PERK	###	<0.0001
		LV-PERK vs. Control	***	<0.0001
**Figure EV5D**	ATP	ANN vs. Control	***	<0.0001
		ANN vs. ANN + LV-PERK	###	<0.0001
		LV-PERK vs. Control	***	<0.0001
**Figure EV5E**	MTT	LV-mCh vs. Control	***	<0.0001
		LV-mCh vs. LV-4R	###	<0.0001
		LV-4R vs. LV-4R +LV-PERK	#	0.0100
**Figure EV5F**	ATP	LV-mCh vs. Control	***	<0.0001
		LV-mCh vs. LV-4R	###	<0.0001
		LV-4R vs. LV-4R +LV-PERK	###	<0.0001
**Appendix Fig S1B**	*3R MAPT*	LV-3R vs. Control	**	0.0016
	4R MAPT	LV-4R vs. Control	*	0.0323
**Appendix Fig S2B**	Control	PA vs. No PA	*	<0.05[a]
	Annonacin	PA vs. No PA	*	<0.05[a]
**Appendix Fig S3B**	pPERK	PA 1 mg/kg vs. Control	*	0.0107
		PA 2 mg/kg vs. Control	**	0.0051
		PA 5 mg/kg vs. Control	**	0.0083
	pNRF2	PA 2 mg/kg vs. Control	*	0.0436
		PA 5 mg/kg vs. Control	**	0.0062
**Appendix Fig S4B**	NRF2	*NFE2L2* siRNA vs. Control	**	0.0055
		Non-targeting siRNA vs. *NFE2L2* siRNA	**	0.0048

[a]For Newman-Keuls multiple comparison test, the statistics program (Graphpad Prism 7.02) does not output exact *P*-values, only ranges.

References

Bouman L, Schlierf A, Lutz AK, Shan J, Deinlein A, Kast J, Galehdar Z, Palmisano V, Patenge N, Berg D et al (2011) Parkin is transcriptionally regulated by ATF4: evidence for an interconnection between mitochondrial stress and ER stress. Cell death and differentiation 18**:** 769-782

Kuhn PH, Wang H, Dislich B, Colombo A, Zeitschel U, Ellwart JW, Kremmer E, Rossner S, Lichtenthaler SF (2010) ADAM10 is the physiologically relevant, constitutive alpha-secretase of the amyloid precursor protein in primary neurons. The EMBO journal 29**:** 3020-3032

Stockwell SR, Platt G, Barrie SE, Zoumpoulidou G, Te Poele RH, Aherne GW, Wilson SC, Sheldrake P, McDonald E, Venet M et al (2012) Mechanism-based screen for G1/S checkpoint activators identifies a selective activator of EIF2AK3/PERK signalling. PloS one 7**:** e28568
