# Supplementary material for: PERK activation mitigates tau pathology in vitro and in vivo
Source: EMBO Mol Med. 2017 Feb 1;9(3):371–84. doi: 10.15252/emmm.201606664 (PMC5331260; doi:10.15252/emmm.201606664)
Supplement: Supplementary file 2 — Expanded View Figures PDF [file EMMM-9-371-s002.pdf]

Expanded View Figures

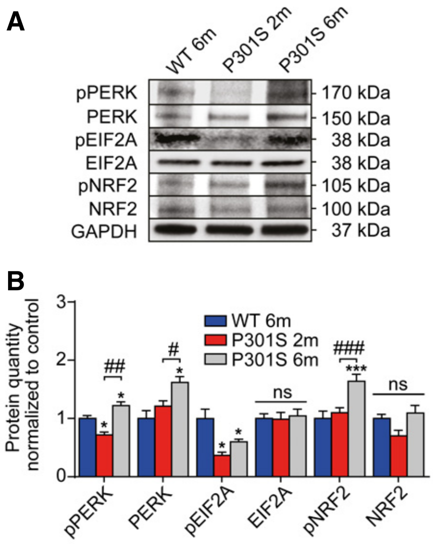

Figure EV1. PERK activity *in vivo* in the mouse model.

A Western blots of whole-brain extracts of mice (wild-type mouse: WT; P301S transgenic mouse: P301S, 2 months of age: 2m, 6 months of age: 6m; $n = 3$ per group) were done with antibodies against phosphorylated PERK (pPERK), total PERK, phosphorylated EIF2A (pEIF2A), total EIF2A, phosphorylated NRF2 (pNRF2), and total NRF2. GAPDH was used as loading control.

B Quantification of (A). Data are mean + SEM. Statistical analysis was one-way ANOVA followed by Student–Newman–Keuls *post hoc* test, *$P < 0.05$, ***$P < 0.001$ versus WT 6m; $^{\#}P < 0.05$, $^{\#\#}P < 0.01$, $^{\#\#\#}P < 0.001$ versus P301S 2m, ns: not significant.

© 2017 The Authors

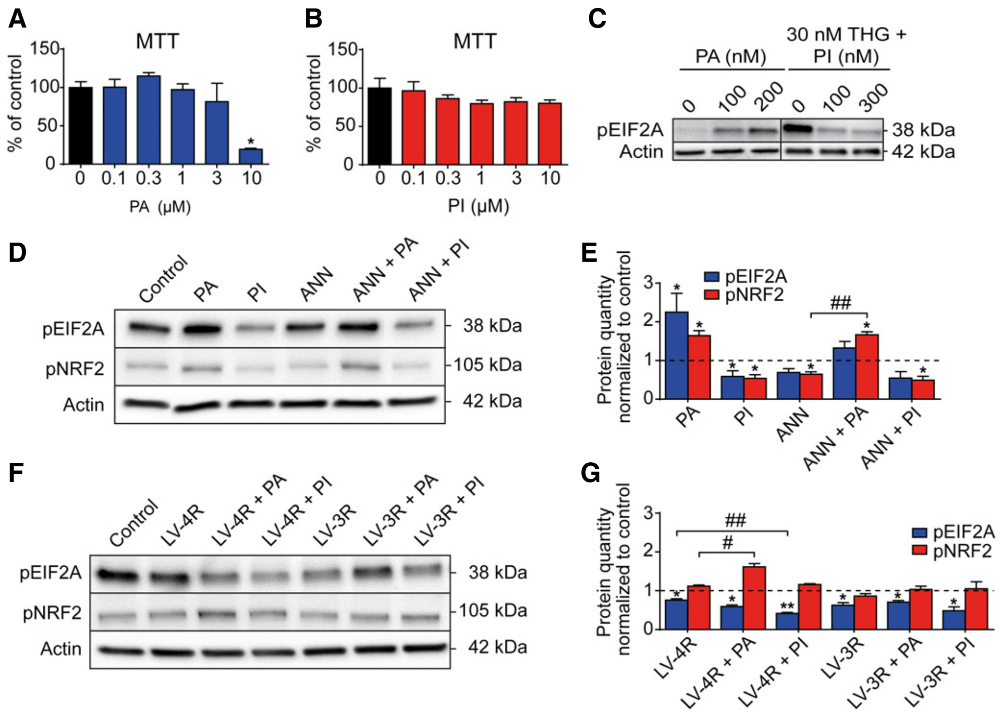

Figure EV2. Activation and inhibition of PERK *in vitro*.

A, B MTT cell viability assays of LUHMES neurons treated with different concentrations of PERK activator (PA, A) and PERK inhibitor (PI, B) for 48 h (*n* = 4 per condition).

C Western blots for phosphorylated EIF2A (pEIF2A) of LUHMES neurons treated with different concentrations of PA, or with the ER stress inducer thapsigargin (THG) and two different concentrations of PI. Actin was used as loading control.

D Western blots for pEIF2A and phosphorylated NRF2 (pNRF2) of LUHMES neurons treated with PA (200 nM), PI (300 nM), or annonacin (ANN; 25 nM), or a combination thereof. Untreated neurons were used as control.

E Densitometric analysis of Western blots as shown in (D) (*n* = 3).

F Western blots of pEIF2A and pNRF2 of LUHMES neurons treated with lentiviruses encoding 4R (LV-4R) or 3R (LV-3R) tau, and PA (200 nM) or PI (300 nM) for 48 h. Untreated neurons were used as control.

G Densitometric analysis of Western blot as shown in (F) (*n* = 3).

Data information: Data are mean + SEM. Statistical analysis in (A and B) was one-way ANOVA with Tukey's *post hoc* test, in (E and G) two-way ANOVA with Tukey's *post hoc* test, paired Student's *t*-test for comparisons to control. $^{*}P < 0.05$, $^{**}P < 0.01$ versus control, $^{\#}P < 0.05$, $^{\#\#}P < 0.01$.

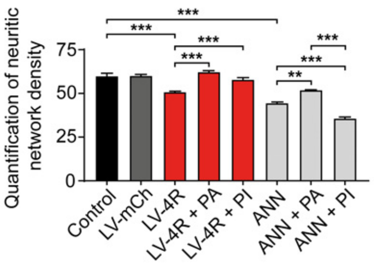

Figure EV3. PERK activator treatment protects neurite network.

Neurites were traced using the ImageJ plugin "Simple Neurite Tracer" by Mark Longair (http://fiji.sc/Simple_Neurite_Tracer) using bright-field micrographs as shown in Fig 2E. Neuritic density was measured in *n* = 5 experiments per group on a scale of 0 (no neurites) to 100 (total surface area covered with neurites). Data are mean + SEM. Statistical analysis was by one-way ANOVA with Tukey's *post hoc* test; $^{**}P < 0.01$, $^{***}P < 0.001$.

 © 2017 The Authors

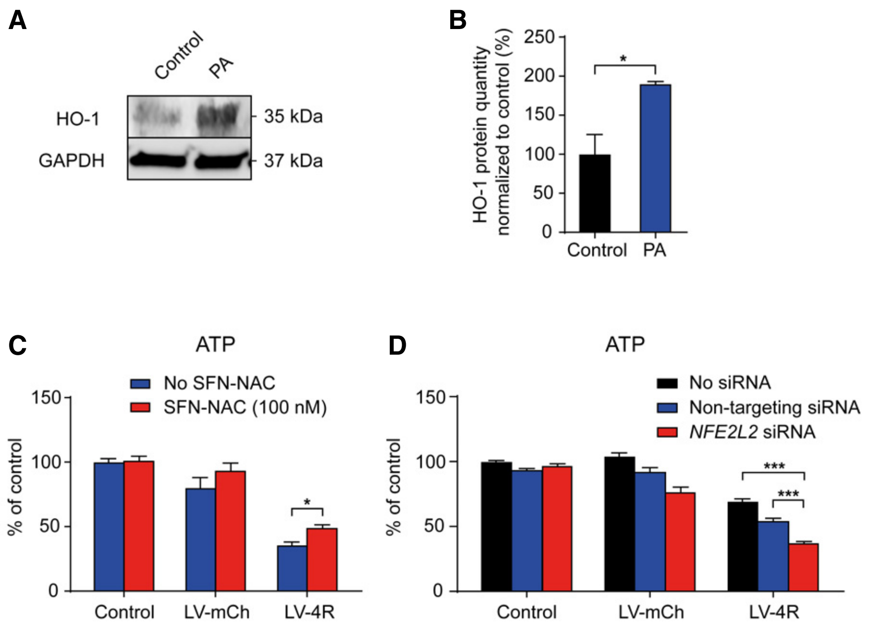

Figure EV4. The PERK activator acts via the PERK-NRF2 pathway.

- A Representative Western blot of LUHMES neurons treated with the PERK activator (PA, 200 nM) using an antibody against heme oxygenase-1 (HO-1); a GAPDH antibody was used as loading control.
- B Densitometric analysis of Western blots described in (A) (n = 3), normalized to the loading control and to untreated control cells.
- C ATP assay of LUHMES neurons either left untreated (control) or transduced with lentiviruses to overexpress mCherry (LV-mCh), or 4R tau (LV-4R) and treated without or with the NRF2 activator DL-sulforaphane *N*-acetyl-L-cysteine (SFN-NAC; n = 6 per group) demonstrates that NRF2 activation protects against 4R tau-induced toxicity.
- D ATP assay of LUHMES neurons either left untreated (control) or transduced with LV-mCh or LV-4R, and with non-targeting siRNA or siRNA targeting the NRF2 gene *NFE2L2* (n = 4 per group), demonstrates that NRF2 downregulation aggravates 4R tau-induced toxicity. Silencing efficacy of NFE2L2 siRNA was demonstrated by Western blot (Appendix Fig S4).

Data information: Data are mean + SEM. Statistical analysis in (B) was Student's *t*-test, in (C, D) two-way ANOVA followed by Fisher's LSD or Tukey's (D) *post hoc* test; $*P < 0.05$, $***P < 0.001$.

© 2017 The Authors

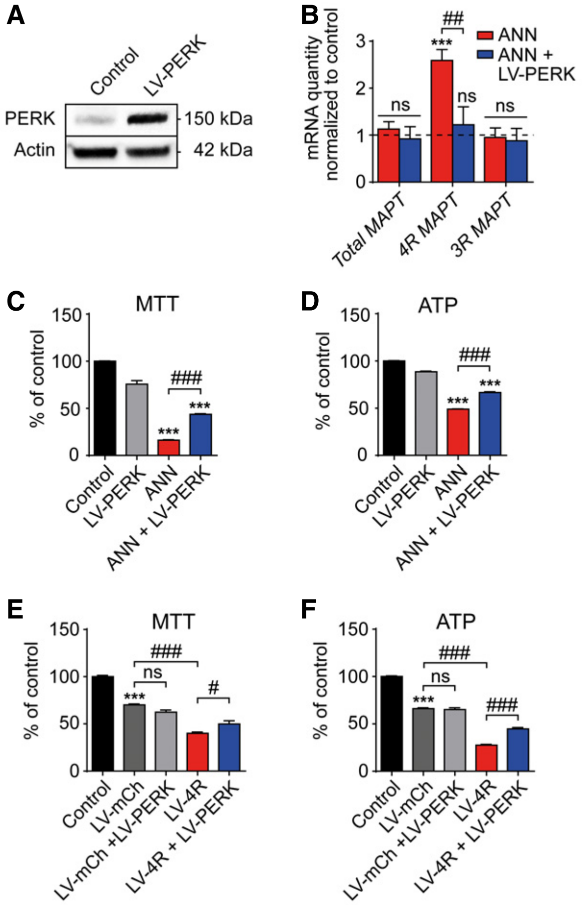

Figure EV5. Neuroprotective effects of PERK overexpression *in vitro*.

A Representative Western blot showing PERK overexpression in LUHMES neurons transduced with mCherry lentivirus (control) or transduced with PERK lentivirus (LV-PERK).

B RT-qPCR of total tau, 4R, and 3R tau mRNAs in LUHMES neurons treated with or without annonacin (ANN, 25 nM), and transduced with or without LV-PERK ($n = 3$ per condition). Dashed line shows untreated controls set as 1.

C, D MTT cell viability and ATP assays of cells treated as described in (B) ($n = 12$).

E, F MTT cell viability and ATP assays of LUHMES neurons transduced with different lentiviruses (LV) overexpressing PERK, 4R tau, or mCherry ($n = 12$).

Data information: Data are mean + SEM. Statistical analysis in (B) was two-way ANOVA with Tukey's *post hoc* test and one-way ANOVA with Tukey's *post hoc* test in (C–F); $^{***}P < 0.001$ versus control; $^{\#}P < 0.05$, $^{\#\#}P < 0.01$, $^{\#\#\#}P < 0.001$, ns: not significant.

 © 2017 The Authors
