# Supplementary material for: PERK activation mitigates tau pathology in vitro and in vivo
Source: EMBO Mol Med. 2017 Feb 1;9(3):371–84. doi: 10.15252/emmm.201606664 (PMC5331260; doi:10.15252/emmm.201606664)
Supplement: Supplementary file 3 — Source Data for Figure 4G [file EMMM-9-371-s003.pdf]

Figure 4 G

Anti-p-Ser-202 tau (CP13)

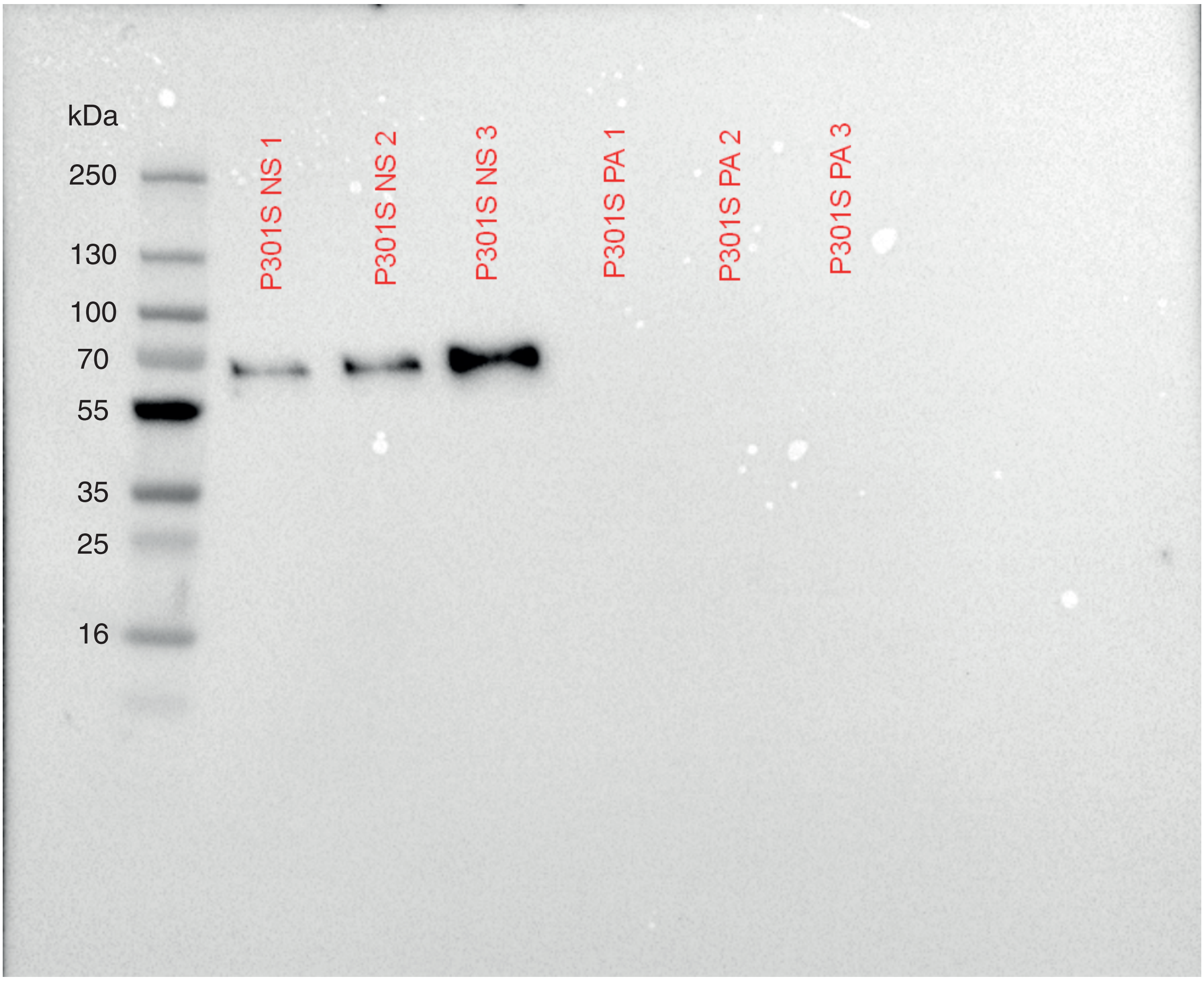

P301S NS= P301S mouse treated with normal saline

P301S PA= P301S mouse treated with PERK activator

Figure 4 G

Anti-p-Thr-231 tau (AT180)

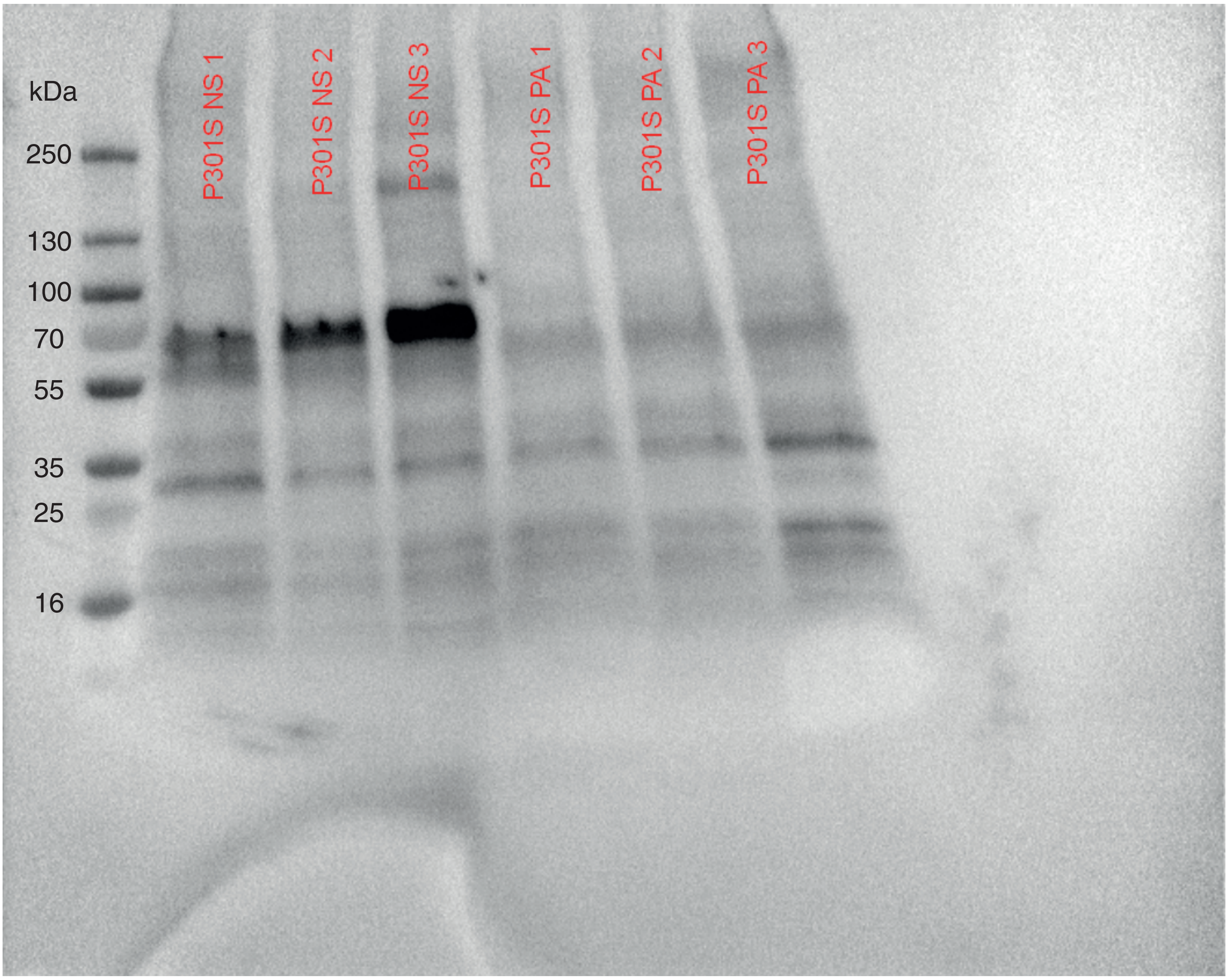

P301S NS= P301S mouse treated with normal saline

P301S PA= P301S mouse treated with PERK activator
